# Contagious Counter-Worlds: On the Idea of an Alternative in German Primary Healthcare

**DOI:** 10.1007/s11013-026-09971-6

**Published:** 2026-02-02

**Authors:** Lucia Mair

**Affiliations:** 1 Department of Social and Cultural Anthropology, University of Vienna, Vienna, Austria; 2 Department of Primary Care Medicine, Medical University Vienna, Vienna, Austria

**Keywords:** Healthcare activism, Alternatives, Germany, Affect, Primary healthcare, Social medicine

## Abstract

This article discusses experimental attempts and imaginaries of interdisciplinary healthcare in alternative group practices, focusing on health activist movements in contemporary Germany and their predecessors in the 1970s-to-1980s, West German ‘Health Movement’. As new forms of working in primary care, these practices stand for reimagining medical practice as a sociopolitical institution, and its possibilities for remaking patient and doctor subjectivities. Both times, this activism is part of broader developments of social-cultural and political change, and pervaded with a stubborn, critical optimism in its potential to foster societal transformations that extend beyond the group practice as a physical place. I explore these experimental projects through Davina Cooper’s (2013) concept of “everyday utopia”, arguing for closer attention to the affects, desires and attachments healthcare professionals, and in particular doctors project onto them. Drawing on utopian studies and health activism literature, I take up Cooper’s distinction between “imagination” and “actualization” to argue how, regardless of its success, experimenting with alternative ways of practising and working together makes current conditions in medicine bearable for those involved.

## Introduction

“I came up with the idea of organizing a counter-world [orig. *Gegenwelt*] during my job interviews, because I didn't feel accepted [in the medical community]. But I soon realized that reforming hospitals from the inside is much more difficult than setting up counter-models from the outside. Nothing is as contagious [orig. *ansteckend*] as productive, convincing new models. And then organizing and doing them: it’s contagious!”

**Figure 1:**
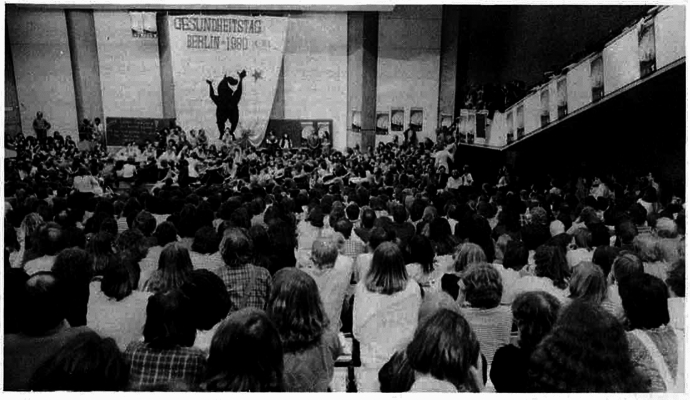
First Health Day, Berlin 1980. Source: Dr. med. Mabuse 1984, 4:34

On my laptop screen, I watch Ellis Huber’s eyes widen as I listen attentively. It is early 2025, and we are in the middle of an interview over Zoom. A retired medical doctor, Huber is an engaging and charismatic storyteller, becoming more animated and gesturing as he describes in more detail this “counter-world”. In 1980, Huber, then a 31-year-old freshly accredited physician from a small village in Southern Germany, became the main instigator of the first ‘Health Day‘ (*Gesundheitstag*) in West Berlin, a countercultural event like none before in German history. Surpassing all expectations, around 12.000 participants—students, doctors, nurses, therapists, patient-activists, social scientists, historians, journalists—sat together in the stuffy, wood-paneled lecture halls of the Technische Universität Berlin to discuss a range of topics, including the history of medicine in Nazi Germany, environmental pollution, disability rights, queer health, abortion rights, antipsychiatry, and hospital working conditions (Deppe, 1987) (Fig. 1).

Organized by Huber and his colleagues of the alternative patient counseling space *Gesundheitsladen Westberlin*, this momentary “counter-world” took up and intensified an unprecedented surge in health activism at the time, and a revived interest in experimentation with alternative models of practising medicine in Germany. “You could feel an energy and power, an atmosphere that radiated to everyone, and I was convinced that this would change the German medical world,” Huber later recalls (Goettle, 2013).

This article is concerned with the possibilities and practicalities of such change. In the years before and after the first Health Day, self-organized, anti-hierarchical, and interdisciplinary group practices emerged across the former Federal Republic of Germany (FRG, West Germany) as a viable yet often precarious countercultural alternative for young healthcare practitioners—an alternative to the dominant, hospital-centric, and curative medicine of the time. Yet like the polyclinics of the former German Democratic Republic^1^ (GDR, East Germany), this model never managed to take hold in the German health landscape in the decades since, for reasons I will explore below. Nevertheless, over the last fifteen years a growing network of health activists under the moniker Poliklinik Syndikat has attempted to re-introduce the concept^2^ to neighborhoods in cities across Germany, and to the medical community at large. It shows a surprising communicability (Briggs, 2005, 2024) once more, being “contagious” not in a biomedical but a social sense: Young healthcare workers are moved, excited, affected by this history and the possibilities this concept seems to afford. By comparatively analyzing these alternative, collaborative medical practices, or health centres, during the health activist movement of 1970s–80s West Germany and among young health activists now, I ask: What is it that makes visualizing, and possibly concretizing, these “counter-worlds” in medicine “contagious,” specifically for some medical doctors? Keeping its multiple meanings in mind, I hold on to the idea of ‘contagiousness’ offered by Huber to think through the “prediscursive flow of contagious affect, feelings, and emotions” (Sampson, 2012, p. 3) that renders some, but not others, susceptible to the transferrence and solidification of this concept, between people and across time. Complicating questions of contagiousness in a narrow, biomedical sense of unidirectional infection, this makes room to also consider how things flow, when they might be “incommunicable”, and why (Briggs, 2024). I argue that for some, these group practices work at the register of what Davina Cooper terms “everyday utopias,” whereby health professionals and patients alike “engag[e] with spaces, objects and practices” in ways that are “oriented to the hope, desire and belief in the possibility of other better worlds” (2014, p. 3). More specifically, I draw on Cooper’s distinction between “imagination” and “actualization” to attend to the question in which ways the realization of the transformative aspirations behind this idea *matters*.

As I show below, attachment to these aspirations is shared between health activists, neighbors, patients, relatives, politicians, insurance providers, architects, and others. In this article, though, I focus on those providing healthcare—employed or not—who are involved in imagining and actualizing these places as spaces where medicine is done “otherwise” (Kehr, 2020; Olufemi, 2021; Povinelli, 2014), and where “new ways of addressing and responding to human misery are worked out” (Scheper-Hughes 1992: p. 215). Considering these “new ways” can engender experimentation with place, with institutional setting, and with the creation of such “counter-worlds”, and puts this article in conversation with other “concrete attempts” of a liberation medicine as invited by this special issue (Führer & Vorhölter, 2025; see also Aragón Martín, 2025).

I pay particular attention to the figure of the physician, arguing that the concept of the group practic forms an affective, exterior infrastructure that allows physicians to come to terms with broader shifts regarding expectations, limits, values, and responsibilities in their profession at different points in the history of now-unified Germany. The group practice, so I contend, is an attempt to respond not only to some of the misery experienced by patients in majority poorer neighborhoods with patchy health and social infrastructure, but also to the misery experienced by disillusioned, overworked healthcare practitioners in Germany’s changing healthcare landscape. Acknowledging this duality contributes to a more honest and nuanced discussion of who experimental practices such as these serve, I suggest. The current national healthcare system in Germany is a mixed public and private insurance system with universal, compulsory coverage. Unlike centrally administered systems such as the National Health Service (NHS) in the UK, it is based on the guiding principle of self-administration: the Bundestag and Ministry of Health provide the regulatory framework, while self-governing structures, including doctors’ associations, health insurance funds, patient organizations and hospitals, work out the rest. This leaves considerable room for negotiation, a fact that has shaped doctors’ working lives, subjectivities, and ideologies for several decades, and which activists have challenged for just as long.

## Theoretical Framework

In their work, both previous and current health activists experiment with counter-hegemonic alternatives to the dominant models of healthcare of their time. They are motivated, on the one hand, by the recognition of a localized lack of sufficient healthcare infrastructure in the urban neighborhoods they service. To these activists, this warrants a specific response. On the other hand, there is something more expansive: an awareness that it is structurally impossible to work within the German healthcare system in the way they imagined and desired to when they decided to go into medicine. They long for “a better way of being and living” (Levitas, 2013, p. 104), specifically, for the possibility of another existence as healthcare professionals for whom better healthcare for patients and more passion for work are equally important demands (Blum & Müller, 1984). This need for alternative ways of relating, working, and existing in healthcare cannot, in this logic, be met within the regular system. Instead it requires the imagination, creation and maintenance of new models.

To account for the different registers at work here, my analysis builds on utopian studies and health activism literature. Drawing on Ruth Levitas’s notion of utopia as desires “to be otherwise, individually and collectively, subjectively and objectively” (p. xi), I analyze these projects as a form of everyday utopia (Cooper, 2014). In doing so, I ask about the medical imaginaries at work here: What “other possible worlds” (Allen, 2015) are dreamed of, realized, and in some cases, left behind, and for whom? Cooper pays particular attention to the daily, material, and affective practices behind everyday utopias as ongoing, speculative, and open-ended projects—projects that themselves prefigure the changes they wish to see (p. 38). Her framework centers the concepts on which utopias are built. For Cooper, concepts are neither mere “ideas or mental constructs through which social life appears,” nor are they sealed off and immutable. Rather, she understands concepts as the “oscillating” movement between imagination and actualization (p. 36), passing through hands and minds in blurry, sometimes inscrutable ways. I found this understanding helpful to approach the concept of group practice through the shape its meanings and practices take for people, at different points in time.

My research is situated in a body of literature on ‘health activism,’ understood as different forms of health-related social action concerning a range of worries and concerns (Zoller, 2005). A broad umbrella concept, health activism is not as common in the social sciences (and outside of academic literature) as activism in other areas, for example ‘environmental activism,’ which conjures a specific imagery and an affective engagement (Kleres & Wettergren, 2017) in a way that the vague descriptor ‘healthcare’ or ‘health’ activism does not. Patient activism has long been a focus of anthropological and social science scholars’ works, most prominently in connection with HIV/AIDS (Epstein 1995), disability (Duval, 1986), and gender and health. Activism by healthcare workers themselves, by contrast, has received less attention. Roughly since the turn of the last century, social scientists have struggled to conceptually delineate forms of health-related mobilization (Landzelius, 2006b). Around that time, several developments converged: restructuring of healthcare in industrialized countries, technoscientific advances in medicine, and a crisis of confidence in science, all of which culminated in a revised “contract” between science and the public, what Kyra Landzelius calls an emergent “politics of vitality” (2006a). Health activism, in her reading, is an expression of this convergence, a convergence that, I would add, we are once again seeing today.

Over the last two decades, many scholars have turned to social movement theory (Frickel et al., 2010; Klawiter, 2004; Wolfson, 2001) to make sense of the health activism landscape, including scholars in (critical) public health, social medicine, and social epidemiology. An oft-referenced typology was proposed by Brown and Zavestoski (Brown et al., 2004), who describe health activism as “collective challenges to medical policy, public health policy and politics, belief systems, research and practice” (p. 52). Later, they proposed an analytical shift from health activism to health *movements*, differentiating between “access” movements focused on improving health service availability, “embodied” movements emphasizing lived experience of illness and disability, and “constituency” movements comprising members of societal groups based on, for example, gender, race, class, disability or sexual orientation (Brown, 2011). Not all such movements hold the collective experience of health and illness at their core, nor necessarily treat it as a source of political drive and solidarity, making the definition useful in a number of contexts. More recently, Susi Geiger and others have shifted back in scale: Geiger proposes the term “healthcare activism” instead of “social movements” to point to the precarious, fragile, and incohesive working mode in which a lot of activism is carried out. Regardless of scale, activists are bound by a “guiding vision” of what Geiger calls the “collective good,” a shared idea of what is moral or right (Geiger, 2021: p. 5; cf. Kehr, 2023; Pushkar, 2019). These aspects also formed the logistical, ethical, and political bases of the activist worlds and work that I encountered during my research, as I will discuss in the following sections.

## Methodology

This study operates from two vantage points, combining historiographical and ethnographic perspectives. I am not a historian, nor is my aim a comprehensive historical account. Rather, I want to outline the concept’s past and contemporary embeddedness to trace its genealogy and contribute to a more nuanced understanding of both the model and its “dissension” from this past (Foucault, 2019, p. 142). Most of my material comprises interview notes, transcripts, and fieldnotes derived from ten months of ethnographic research among health activists in several German cities between 2022 and 2024. During this time, I attended formal and informal meetings, protests, public presentations and conferences, political actions, and celebrations. After two preliminary scoping visits in the winter of 2021 and spring of 2022, I chose one health center and its surrounding neighborhood in a metropolitan area of Germany as my primary field site. From there, I conducted long-term fieldwork as an observant participant (Pine, 2013), following daily routines and practices of health center staff. I sat in on consultations and meetings, followed along on home visits, and discussed with activists how they saw their approach to healthcare reflected in specific situations. In addition to observant participation, I conducted semi-structured interviews with patients and visitors to the health center, with neighbors, health insurance representatives, other researchers, and local politicians. These conversations have informed my analysis throughout, and I focus on them in depth in subsequent articles (Mair, forthcoming).

Over time, activists' engagement with the genealogy of the concept of the interdisciplinary group practice emerged as a salient theme. Using snowball sampling, I interviewed participants from predecessor projects to explore their motivations, experiences, and challenges. These oral histories were complemented by archival research on alternative health projects and analysis of historical publications, grey literature, and artifacts like pamphlets, posters, and zines. Additional sources included newer publications, websites, policy documents, and public health research articles. Informed oral consent was obtained prior to all interviews. All names of persons have been pseudonymized unless requested otherwise. In several cases, interlocutors’ gender has been changed where irrelevant for the analysis of data, to ensure anonymity within this small field. Empirical data was analyzed using qualitative content analysis (Mayring, 2019). The majority of fieldnotes, interviews, and analyzed documents were originally in German. Unless otherwise noted, all translations are my own.

Regarding my positionality, this research is shaped by my role as a “triple insider” (Pushkar, 2024)—as a doctor, a German citizen, and a health activist who shares a strong commitment to health equity with the activists of my research, though our methods differ. Over time, these similarities can challenge and transform analytical distance, and at times have done so. This required ongoing reflection on possible oversights in the dynamics I paid attention to (or failed to), and on disentangling interpretations of my interlocutors from my own, with other scholars doing similar research, supervisors, and interlocutors in the field. To that end, I chose to engage the project with a reparative attitude that takes into account my being affected as a scholar-activist (Birn, 2025; Love, 2010), rather than a purely critical one that pretends otherwise (Fitzgerald, 2017). By taking this route, I hope to voice a form of “comradely critique,” as suggested by Lukas Slothuus (2023) and developed further by Piyush Pushkar, that is both a “political intervention” (2024: p. 60) *and* an attempt to advance “solidaristic relations” between scholars, doctors, patients, and activists (ibid.). My hope is that what is intended as affirmative critique will be received in the spirit it is given, one of curiosity, appreciation, concern, and hope.

## Medicine, Set in Motion: Health Activism in 1970s and 1980s West Germany

“For us, the Health Day of 1980 was not only hard work but also an emotional experience, a friendly and warm counter-world (orig. *Gegenwelt*) for a few days that made everyday life more bearable and our own prospects more hopeful again. At the same time, it was an impulse to think about ourselves, our professional identity or perspective as doctors and the role of medicine as we have known it up to now. Medicine was set in motion by and with the first Health Day.” (Huber & Lundt, 1991)

If we want to better understand the affects and effects surrounding this “counter-world” described by physician-activists Ellis Huber and Stefan Lundt ten years later, and how its utopian aspirations resonate in current activism in German medicine, we need to consider the past. What dominant model of healthcare in Germany did activists counter back then, what were the alternatives they put forth, and how do they resonate now?

As I will show, today’s alter-models in the primary care sector point to the local permanence of a broader social criticism that took hold in Germany in the late 1960s to mid-1980s in the so-called ‘Health Movement’ (*Gesundheitsbewegung*). Emerging at the interface of the West Berlin squatter scene, feminist women’s health movement and alternative health spaces, the Health Movement figured a momentary “counter-public” advocating for patient-oriented, holistic, and democratic healthcare. The movement culminated in several large-scale events, including the 1973 ’Marburger Kongress’ and later, the ‘Health Days’ (*Gesundheitstage*) of 1980 (Berlin), 1981 (Hamburg), 1984 (Bremen), and 1987 (Kassel). Both then and now, institutional, epistemological, and political critique of medicine came together in ways which reflect the mood of their time, and it is precisely the activism around the Health Movement which, I argue, makes possible the imagination of the experimental social clinics of today.

The Health Movement emerged at the tail-end of a period of stability in the West Germany of the 1950s and 1960s. In this phase of post-war economic growth, the idea of the German welfare state was associated with reforms rapidly expanding pension, health insurance, and other social benefits (Lindner, 2004). Driven by increasing prosperity, the number of health service providers and the range of treatments offered grew significantly. Towards the mid-1970s, however, this was followed by a phase of cost containment wherein the reform of the welfare state took on a different significance, now emphasizing austerity measures and cost limits. Many of the problems of the German healthcare system of today can be traced back to this past, including those to which today’s activists respond (Porter & Guth, 2012). The West German system in particular changed profoundly during this time: Care was increasingly marketized, technologized, and specialized, and it increasingly took place in hospitals rather than small practice settings. In East Germany, meanwhile, socialist property relations fully subordinated health issues to the state and its decision-making structures. Prevention and living and working conditions were a priority, leading to the construction of a network of interdisciplinary polyclinics and state medical practices, the ‘Ambulatorien.’ Healthcare professionals in these polyclinics were employed by the government on a fixed income. Physicians were expected to work together with other healthcare professions, and to come to medical decisions independently of economic considerations. Notably, the Eastern and Western systems had opposite effects on the historically steep hierarchy between different professions in healthcare.^3^ Physicians in East Germany, reluctantly and gradually, embraced the idea of working cooperatively in polyclinics, though they were initially worried about losing their historically highly valued (professional and fiscal) independence in private practices. In West Germany, most physicians practiced alone—and still do so today. Each of these histories has affected today’s imaginaries of the model, and they matter when approaching it anytically.

These developments in a divided Germany coincided with (and contributed to) a global “second wave” of social medicine (in particular in the Latin American Social Medicine tradition, see Carter, 2025). The World Health Organization (WHO) famously promoted a shift away from hospital-centrism towards a more social and community-based approach to healthcare in its Alma Ata declaration (1978) and the Ottawa Charter (1986), affirming the role of the primary health care (PHC) sector in the goal to attain “health for all”. Health in a broad sense was re-affirmed as a basic right, and in a short-lived and utopian push for universality, WHO actions and programs reoriented to promote this right (Kehr et al., 2023; Pentecost et al., 2021).

Medicine had again become politicized, and politicizable. In Germany and elsewhere, this period proved fruitful for experimenting with the sociospatial arrangements of medical care, with the imagination of “counter-models,” and their enrolment in other efforts towards social justice. Group practices emerged as their main form: in East Germany, as polyclinics and Ambulatorien; in West Germany, as alternative group practices.^4^ The Health Movement's main criticism—of the understanding and methods of biomedical, curative medicine—arose from a broader, critical understanding of society at large, reflected at the time in other grassroots, post-1968 protest movements, including the women’s movement, the peace movement, and environmental activism. This criticism was broadly split into two, at times overlapping, factions: on the one hand, those who were skeptical of rapid technologization, institutionalization, and the growing influence of the pharmaceutical industry in medicine, and who promoted more naturopathic, homeopathic, and sometimes esoteric healing methods; on the other hand, politically active healthcare professionals, including groups like *Kritische Medizin* (‘critical medicine’), which formed especially among students in almost all West German medical universities (Deppe p. 178)^5^. *Kritische Medizin*, as sociologist Hagen Kühn notes, cannot be understood as a “consistent theoretical concept like 'critical psychology,' but rather a collective term for left-wing, initially mostly Marxist-oriented criticism of medicine and the social conditions of health and illness” (2012, p. 417). These groups printed their own leaflets and zines in DIY fashion,^6^ educating local publics on health-related issues and medical history. They were crucial for the formation of the Health Movement, and the emergence of the model of group practice, as Uli—a physician-sociologist—recounted in one interview:

“There were ‘critical’ students—once they were students, then they began residency training. And after, they usually had to set up their own practice [and started to ask]: How can we maintain the communication that exists in hospitals, where people can inform and advise each other, in our practices? How can we continue to maintain the kind of teamwork that was possible in hospitals but is no longer possible today? The answer: we do group practices.”

With a physician’s assured confidence, he tells me that it was these “frustrated doctors” who experienced hierarchy and interdisciplinarity in hospital medicine who “make the difference, or who provide the steam, the motivation” to formulate these ideas for alternative practices. The trajectory he describes here—from individual experience, to an affective connection with others, to the possibility of “imagining” and possibly later “actualizing” a form of everyday utopia—speaks to the works of feminist theorists, such as Clare Hemmings, who postulate that “in order to know differently we have to feel differently” (2012: p. 150; cf. Ahmed, 2004; Berlant, 2007; Cooper, 2014; Probyn, 2004). The disjointed feeling that “something is amiss” (ibid.)—the stress, frustration, and alienation Uli and others of this generation felt in an increasingly technocratic, singularizing, hospital-based West German healthcare system (Schmidbauer, 1978)—created an affective, embodied, and “critical dissonance” (Hemmings, 2012: p. 151), and a political impetus that opened up new ontological and epistemological possibilities. Feeling allowed for the imagination of other forms.

In struggling with these experiences, ‘critical’ doctors at the time of the Health Movement also challenged mechanisms of their own clinical subjectivation within a restrictive system (cf. Holmes et al., 2011; Mukhopadhyay, 2016): What they imagined for their work was “not a technically perfected apparatus that cuts the body and illness into mg% units, but a medicine that treats people holistically, somatically and psychologically” (Blum & Müller, 1984, p. 56). Their attempt to craft another clinical gaze for the self (Stonington, 2011) directly countered four core structural features of the orthodox, West German healthcare system after 1955: somatization, fragmentation, individualization, and de-politicization. In 1955, a legal change had introduced the renumeration of individual services in office-based and hospital care (continued in a related form until today as Diagnosis Related Groups (DRGs)). Before 1955, West German doctors were paid according to a per capita flat rate, a fixed amount for each patient treated regardless of the extent of care. Now, doctors had greater incentive to expand and make use of a broad range of their services. They increasingly specialized, which for patients meant they were treated according to organ-specific pathology, in distinct places and in interactions with different people. The same 1955 law cemented the near monopoly of individual, registered doctors in individual practices to provide outpatient care in West Germany, de facto forbidding other structures such as the insurance-owned *Ambulatorien* of the 1920s. Other health professions were devalued, while physicians’ near-total power over the ambulatory sector was made permanent. In return, statutory health insurance physicians waived their right to strike (Ahls et al., 2018; Busse et al., 2017).

Some young physician-activists did not want to conform to those changes. An excerpt from the written report after the first Health Day 1980 in Berlin reads:

“All of this [i.e.: increasing technologization, time pressure, bureaucratization; LM] no longer corresponds to the ideas and ideals that were the reasons why we became helpers/healers. It has to change. Because when dissatisfied, it's hard to live and work well. The driving force for change is therefore a selfish one: we want to gain affirmation/satisfaction through our work. Namely the affirmation that we can help, that we can truly heal.” (Burkhart and Mindel 1981: p. 7)

With this critique, they acknowledged the extent to which doctor-patient and colleagueal interactions in medicine can transform subjectivites and social relations, beyond the individual patient body (Cooper, 2015; Livingston, 2012; Taussig, 1980). At stake were not only personal disappointment and disillusionment after joining the medical field; these activists were grappling with the highly politicized question of what it means to “truly heal,” and what “*echtes Arzttum*” (“true physicianship”) entails (Lindner, 2004, p. 85). At that time, this had already been a point of contention in Germany for several decades: the question of the right way of doing medicine, of *being* a member of the medical profession, of embodying medicine itself. The independence of physicians was part of those negotiations, defended by some as a core “value of Western society” (ibid.). As the first post-WWII generation trained into a profession that had barely publicly and collectively acknowledged its role in the Holocaust, the Porajmos, the murder of queer people and people with disabilities—until the first Health Day, where this was the main agenda point—this question was especially pressing, and for many, personal and painful (Czech et al., 2023; Forsbach, 2011). The model of the group practice seemed to provide a counter-infrastructure for practicing a form of “true physicianship” that aligned more closely with how these activists saw themselves:

“For a long time, our hope for an identity of life, work and politics was focused on group practices. What was always torn apart in traditional ideas of work and politics seemed to be conveyed here in a holistic life model [orig. *Lebensentwurf*].” (Blum & Müller, 1984)

This quote underlines the complex enmeshment and daily effort of aligning professional role, moral selves, and socio-political aspirations for these activists. In this way, the model of the group practice transposed an imaginary ideal of one of the central tenets of social medicine—holistic medical care—onto the lives of doctors themselves. The goal was to counter the individualization, fragmentation and de-politicization of ambulatory care physicians that had been formalized in the 1955 changes, including the relinquishment of their right to strike. This striving for holism was not unlike other countercultural projects of the time (Jütte, 1996; Reichardt, 2014), but in the case of medicine it was more clearly tied to questions of moral subjectivation and its attendant practices.

Small, alternative group practices existing in the margins of the spectacle of the Health Days thereby can be viewed, as my findings indicate, as the epitome of a “contagious counter-world.” Reflected and actualized in this model were transformative beliefs, desires, and utopian aspirations of medical practitioners concerning their daily working lives—maybe not altogether surprising, given the era. These desires however extended beyond the clinic, beyond discomfort with power, professional rules and expectations, to encompass the way practitioners, individually and collectively, moved within and related to their social worlds, and the changes they imagined for medicine and society at large. Speculating with new medical forms at the time was neither only about doctors’ imaginaries of a different work-life nor only about patient care; rather, it was borne out of a strong sense of mismatch between self-image and the infrastructure available for this care, and it connected with a deeper engagement with the nature and responsibility of the medical profession. In this sense, affective experience and attempts to build anew the institutions one imagines were always entwined.

As intentional misfits in medicine, however, these experiments ran into trouble from the start. Invigorated by the Health Day, group practices and so-called “Health Stores” (*Gesundheitsläden*; doctor-less patient counseling services) “sprang up like mushrooms” all over West Germany (Kastenbutt & Westen, 2003: p. 128). Group practices were structured around at least one registered general practitioner carrying fiscal and administrative weight, and could include physiotherapists, medical technicians, social workers, psychologists, and so on (Gesundheitsladen, 1980). Bound by conviction that a more egalitarian way of living and working in healthcare was possible, most groups tried to pool income and shared decision making power. In practice, working there meant a “colleagual atmosphere” but also “more stress and less money,” at least for physicians, summarized internist Friedrich Kater (1981: p. 230) from the *Gesundheitszentrum Gropiusstadt*. This West Berlin group practice opened in 1976 after a five-year planning phase. Kater later recounts: “The group was only able to withstand the heavy, psychologically and time-intensive toll over a long period of time because there was an imminent chance of realization [of the project]” (p. 225). Few of their doctors had worked outside the hospital before, and knew little of the material realities of the renumeration system in which “you have to work extraordinarily hard and fast" (p. 228) to achieve a sufficient number of patient visits to survive financially. Shared patient files—an anomaly at the time—fostered transparency and synergies, but also insecurities and dissidence. Pooled wages were abolished after the first year. Power imbalances posed an insurmountable problem. Writing under anonymous authorship in the same publication, colleagues of Kater summarized that “only the white coat was taken off,” while internalized professional hierarchies persisted (p. 233; cf. Rickmann et al., 1982 for a similar account). Their disillusioned conclusion after several years was that the model could never be a *true* alternative in the primary care sector since it “can’t fight against the system but rather has to exploit it” to survive (ibid.). Paradoxically, they warned, the group practice risked becoming the “perfect medical apparatus” (ibid.), a useful alibi to mask structural service gaps in underserved areas through extraordinary personal engagement of activists and patients alike. In this apparatus, the “essential moments” (Elgeti, 1977: p. 122)—mutual support, fair pay, relationality—which made the concept meaningful and ultimately “contagious” got lost. While the Health Movement dwindled away after a few years, and the majority of group practices have not survived, the ideas, affects, and histories behind them have, as I will discuss in the following section. This complex now figures an integral part of the infrastructure making today’s utopian attempts possible and imaginable, and enables today’s activists to conceive of alter-models once more.

## Concrete Places? Re-emergence of a Model in German Cities of Today

In the 1960s to 1980s, as I have traced, primary care emerged as a fruitful field for health activists, practiced as “a form of social medicine, and social medicine as a social critic” (Pitti, 2021: p. 307). In the newly unified Germany of the 1990s and early 2000s, the discipline retreated somewhat into the background of a national health system increasingly organized within neoliberal logics. General medicine came to be provided by mostly single physicians in individual, self-owned, and economically independent practices (as it still is). Most of the polyclinics, the state-run ambulatory health centers of former East Germany, were closed down. The Health Movement largely disappeared from view, mired in internal disagreement between members of differing interest groups and facing ongoing opposition from the more conservative, West German medical establishment (Göbel & Schagen, 1982).

Roughly for the last fifteen years, however, a second shift in primary care has been underway. In Germany as in other geographic contexts, social medicine principles are experiencing a comeback. The central position of primary care and general practitioners as a frontline entrypoint to an overburdened health system is acknowledged once again in the German public, not least since the COVID-19 pandemic. Part of this shift is the (re-)emergence of new roles and modalities, e.g. community health nurses and ‘health kiosks,’ doctor-less patient counseling services mirroring the ‘health stores’ of the 1980s (Köckler et al., n.d.). Many of these efforts are intended to cut costs by streamlining patient navigation through the system and encouraging longitudinal patient “management” tied to one institution and physician. The overall aim is the systemic prevention of unnecessary, resource-intensive patient visits. Against these logics of cost-efficiency, a small wave of opposition has formed that expresses itself once more in the experimentation with alternative models of providing care in underserved urban areas:

“I think it's extremely essential to create concrete places. Places with walls, with windows, and doors. So that we can experiment, and so that we can fail, too.” (Marlene, November 2023, fieldnotes)

Marlene, a general practitioner in her late thirties, raises her arms. Sitting on a chair in a circle with others, she draws lines in the air, waves her hands, trying to outline a rudimentary house. I sit in the outer circle with about 25 other audience members, listening to a discussion between Marlene and four others—a doctor, a social worker, a nurse and a sociologist—as part of a symposium on health activism in a mid-sized German city. Perhaps unknowingly, Marlene echoed Friedrich Kater’s sentiments about the taxing early stages of the *Gesundheitszentrum Gropiusstadt,* only, forty years later: She is part of a growing, nationwide network of young health activists working to create a “counter-world” once more by reviving the old idea of the group practice. They go by the moniker *Poliklinik Syndikat*, in reference to the East German predecessor clinics of the same name.

Their goal is to open “social solidarity clinics” as a form of interdisciplinary, neighborhood-based community health centers based on a bio-psycho-social model of health, with a focus on prevention, participation as well as environmental, political and structural health determinants. Specifically, they enter what they term “structurally weak” (Poliklinik Syndikat e.V. n.d.), historically working-class and immigrant neighborhoods with few available health and social services for locals. At the time of writing (Spring 2025), around twenty groups exist in different cities, comprising doctors, nursing staff, physiotherapists, and psychologists, as well as social workers, artists, political scientists, IT specialists, and architects. The individual groups and the network are tightly organized and at different stages of envisioning, concretizing, or already running a health center. Their ambitions work on three registers: making comprehensive, discrimination-free healthcare accessible to the individual patient-body; making their own working conditions meaningful and non-hierarchical; and fashioning a “new, solidarity-based healthcare system” for the German public as a whole (Poliklinik Syndikat, 2025). Healthcare, in their view, is inherently a question of politics, and a critical arena to change broader societal values and functionings. Echoing the ambitions of the Health Movement, the network refers to itself as a “movement” that has “accepted the fight for social infrastructures,” as one physician described it in an interview, in parallel to healthcare workers’ struggles against spending cuts and privatization in other countries (e.g. Spain, UK, Greece, France, or South Korea).

Many of the activists with whom I spoke described a similar trajectory: often they had been part of other health activist projects, for example on migrant health, hospital labour conditions, planetary health, LGBTQIA+ rights or abortion access. Two medical students, Sofia and Valentin, separately illustrated this point: Both initially founded and led *Kritische Medizin* groups at their respective universities, similar to the predecessor groups of the 1960s–80s, through which they questioned norms, hierarchies, and exclusions in the German healthcare system. After learning about social solidarity clinics through friends and public events, each told me how excited they had been to learn of this model as a promising answer to many of their concerns. Each of them decided to commit fully to work towards setting up a clinic, in their free time, and considered working there themselves later. Both left their respective *Kritische Medizin* groups. This spontaneity, the willingness to “just start somewhere” and “let oneself get euphoric about things” was named by Christian, a physician in one of the existing clinics, as a core charasteristic of longterm members of their network. I found this notion of imagining an alternative, potentially better configuration of medicine in this pre-figurative phase to be most powerful and “contagious” among a specific type of mostly young, well-educated, often White students and healthcare workers: For them, the concept seemed to stir, intensify, and channel an already-existing utopian impulse (Bloch, 1986) whose origins lay in an awareness of incompleteness, of something feeling not quite as it should. To some, it manifested as a sense of foreboding regarding one’s own future in the existing healthcare system. Medical student Sofia described these feelings as

“a sense of powerlessness and despair about becoming part of this system myself. Not just being a witness, but also being an active part of the problem […]. Since I've been studying medicine, more and more people around me have been telling me their stories from the medical field, and ninety percent of them are just a disaster. […] It's not that I... had such a negative experience myself, that I felt I absolutely had to go into medicine to change that, or something, it's not that. It's more [realizing], God, I don't want to reproduce that.”

Sofia describes a growing awareness of the double bind of physicians who simultaneously hold immense power vis-à-vis individual patients but feel powerless themselves within historically grown, confining structures (for description of a similar experience in psychology, cf. Alemohammad, 2025). In her work on abolition medicine, Carolyn Sufrin (2025) terms this “complicity consciousness,” an awareness of one’s wrongdoing and upholding of harmful structures in a way that is entwined with the reimagining of systems. It is precisely when this awareness blends with the aforementioned permission of medical professionals to “let” themselves “get euphoric” that they become susceptible to the “contagious” idea of the counter-world in the first place, while others do not. As was the case with the previous activist generation, affective experiences of sadness, disillusionment or anguish about the possible implications of one’s professional role—as a figure capable of doing harm—are tied to the utopian imagination of another form, and to attempts to actualize it. In recent years, groups have been formed, members found, networks established, internal trainings held, and blueprints for how to open such clinics written and freely distributed online. Alongside sharing knowledge and practical advice, members have provided each other throughout with reassurance, encouragement and hope that actualization is possible, and soon. In a sense, these shared feelings, beliefs, and histories have formed the necessary affective scaffolding for the forceful re-emergence of the idea and concept of the group practice. Considering this scaffolding brings us closer to the questions of who this idea is for, and what it does for whom.

In part, these fascinations and attachments are undergirded by the concept’s strength as the structural antithesis of the current cultural and legal framework of primary practice in Germany. As described above, primary care physicians in this system usually work alone or with few colleagues of the same discipline, are fiscally responsible for generating their own profit, and do not routinely interact with professions outside of medicine. Conversely, the social clinics manufacture an interdisciplinary work environment which, although patchy and flawed, facilitates a sense of relationality and responsibility among colleagues that can be otherwise elusive. Sofia told me of her hopes for her local group to “fully understand itself as a collective,” whereby everyone “looks out for each other … and that this results in a place where we ... well, where people and health are addressed in such a way that there is as little hierarchy as possible and as much [interdisciplinarity] as possible.” For Sofia, imagining this idealized working life enabled her to go from theoretical to practical engagement with questions of health injustice, although she, too, found it easier to do so on the concrete exterior provided by the utopian “counter-world”: The ‘Critical Medicine’ group she left had aimed to change healthcare from within existing institutions, through critical education and advocacy, but proved insufficient for what she longed for.

Esther, a physician in her late 30s who works in one of the clinics part-time, similarly notes that it is especially the days when she has “had a lot of contact with other departments”—psychologists, community health nurses, social workers—that also tend to be “the days when I just go home satisfied, no matter how busy it was, because it just feels right and meaningful.” Nowadays, as in the 1980s, it is these very aspects that are both most intriguing and significant for health activists *and* structurally inhibited by mundane practicalities within the legal framework of primary care in Germany. Two examples with everyday, practical consequences illustrate this point. First, there is currently no billing code for interdisciplinary consultations. Instead, they need to be refinanced through a higher number of orthodox examinations in order to generate enough profit to balance the budget. The paradigm of “extraordinarily hard and fast” work, already present in the group practices of the 1980s, has become more pronounced amidst general work intensification in Germany’s medical sector over the last decades. Second, there is currently no patient records software for sharing thoughts and findings that can be accessed by more than one profession—something that is essential for interdisciplinary work. This leads to complicated, ad-hoc, and informal workarounds in the social clinics to exchange information on individual patients without violating data privacy and confidentiality. Though seemingly minor, these two examples have a significant effect on quotidian routines, interactions, and relations. As a consequence, activists must also share a “kind of pragmatism,” as physician Christian notes, in addition to the utopian impulse and an “undogmatic left-wing attitude.”

The structural reliance on medical doctors within these projects also brought a precarious velocity, and hindered efforts to flatten hierarchies. Dilan, a clinic employee with a nursing degree, pointed out that “many, or not many, but a few [of us] have not studied, or studied other things than what [many of the decisions] are about. And … yeah, I can't acquire the knowledge quickly enough to then participate in decision-making." These decisions varied in weight: from which towels and plants to buy, to the best messenger sevice for internal communication, to which theoretical frameworks and traditions to use to explain the causes of social and health inequity, to the formulation of project aims (e.g. referencing Breilh, 2023, or Waitzkin et al., 2001; cf. Harvey et al., 2022). Likewise, medical doctors shaped temporalities: The ethos of “simply running with it” that one of the physicians mentioned as a requisite for members of the collective mirrored the confidence, impatience, and sometimes lack of empathy trained into medical professionals from early on (Holmes, 2025). Such implicit hierarchies were sustained through words and actions that were more subtle than the harshness we know from hospital floors, underscoring how difficult it is to recognize and resist a trained clinical gaze that is embodied and enacted both on the patient, on colleagues, and on the self.

Since its begininings, the concept of the interdisciplinary group practice has rubbed against the edges of German medical bureaucracy in the current socio-politico-legal context. This leads to friction, tiredness, and increasing disillusionment, as Jörn, a former clinic employee, alluded to at the end of our conversation, with some hesitation:

“Well, the initial vision was that we would provide better care and have better working conditions for ourselves. That was the vision at the end of the day. And I thought both were good, I still think both are good. [Pause] We don't have better working conditions, we are completely exploiting ourselves in what we are doing here”.

Jörn had been involved in the project for several years as a medical technical assistant, in salaried and volunteer roles alike, before leaving, in part due to the high workload. As my data makes clear, he and his colleagues struggled to balance competing ambitions and demands inherent in the concept: how to run a health center, identify and engage a “community,” care for the collective, and nurture their individual, moral, and political selves. Yet almost everyone I asked—including physicians, social workers, nurses, and psychologists—asserted that they would not want to go back to working in regular healthcare. Jörn, too, assured me that he wanted to remain a volunteer member of the local collective and nationwide network, bringing more social clinics into being.

While the utopian imaginary most held onto before working in the social clinic did not match its messy, complicated actualization, this barely affected activists’ strong belief in and attachment to the concept: It maintains and nurtures its appeal, changes shape as needed, and sticks with people without, for the moment, needing to function in its own logic nor fulfill its own promises. Approached within this specific, German history with all its affective weight, its hopes and frustrations, I suggest that the concept derives this strength as a figuration of an Otherwise (Povinelli, 2014), a utopian aspiration of a “counter-world” for healthcare workers that anticipates “something more, something beyond and other” to what for them is currently viable and doable within medicine as a hegemonic space of power (Cooper, 2014: p. 4). Its lines of friction are always visible. Yet through various doings—network-building, campaigning, blueprint-making—activists hold on to a horizon of hope and belief in their individual and collective potential to mend those lines, and to move beyond the possibilities of the present.

## Conclusion: The Impossibility of the “Good Space”

As my research has shown, both in the 1970s/1980s and now, different individuals and activist groups re-adapt aspects of the idea of the interdisciplinary group practice across Germany in uneven, partial, and often improvised ways. This brings us back to my initial question: Does the realization of the transformative imagination behind this idea *matter*?

For the moment, as I have demonstrated, the concept is circulating, experimental and, at least for some, “contagious”. As such, it hints at “latent possibilities and potentialities held within” which, as of yet, one “might only glimpse obliquely” (Meek & Morales Fontanilla, 2022: p. 275): The idea is held in abeyance between imagination and actualization. On the one hand, activists are able to “hold or open to liberatory transformation” (ibid.); on the other, they acknowledge its limits in preventing harm, and its own tendency to (re-)produce some of the injurious conditions to which the concept claims to offer an alternative. Following Lauren Berlant, this moment marks an “impasse,” wherein embodied hierarchies are upheld, harmful precarities reproduced, and meaningful and relation-affirming aspects of work unpaid (Berlant, 2011). Yet, this moment is carried by the refusal of activists to abandon their commitment. They continue to anticipate a sociopolitical shift wherein the concept will become sustained, and sustainable, a shift not likely to happen anytime soon because the German healthcare sector is moving in unpredictable directions in the 2020s. These contradictions throughout time underline the fragility, and yet strength of the concept.

At this moment of suspension, any liberating potential the concept might hold, if at all, lies first and foremost in its affective hold: It continues to mobilize young healthcare activists—especially doctors—as a form of an “imaginative affordance” (McClelland & Dunin-Kozicka, 2024) that allows them to see and place themselves in an *other* medicine. Thereby, it makes current conditions bearable (Cooper, 2014), providing momentary relief from a system which, structurally and culturally, grants healthcare workers immense power and responsibility but also causes them discomfort, loneliness, and grief. To them, the idea becomes a vitalizing fiction of horizontality that offers a small niche to imagine, and later attempt, a version of oneself providing care in a way that aligns with personal convictions, morals, and values. At the same time, these “glimmers of possibility” (Mukhopadhyay, 2016: p. 62) bring the provisionality, contingency and open-endedness of any utopian project into sharp relief, within restricting political conditions in a neoliberal, capitalist healthcare system: An “Otherwise, kept at bay,” as P. Sean Brotherton has termed this state in-between “constraint and possibility” in the context of re-structuring of Venezuelan primary healthcare (2025: p. 311).

Today’s health activists in Germany engage with, extend, and amend a specific part of German medical history as an expression of individual and collective affective experience, and as an aspiration across scales—not only for the healthcare system, but also for their own selves, and for society as a whole. Thinking with César Abadía-Barrero, medicine here becomes political imagination in a way that is “not a purely rational or intellectual exercise of ideas” but is rather borne of a specific “epistemology of care” rooted in “shared experiences, history, and materiality” (2022: p. 227). In this case, it is the sum of experience, history, and materiality amid a decades-long transformation of medical practice—as one’s chosen profession—in ways that fundamentally oppose healthcare activists’ beliefs, expectations, and politics. Over time, the concept of an interdisciplinary group practice that is politically and socially engaged has become a form of insistent, stubborn response to the question of “true physicianship,” and to what it means to work in healthcare more broadly. Is this imagination or actualization? Here, it is both: the imagination of an “impossible kind of good space” (Cooper, 2014: p. 3) that, at the same time, animates collective action for social change. It articulates and, in doing so, enacts an alternative version of and vision for lives and subjectivities in the medical space.

## Data Availability

No datasets were generated or analysed during the current study.

## Footnotes

1 From here on and for simplicity’s sake, I will refer to West Germany and East Germany as parts of reunified Germany that correspond to the former FRG and GDR.

2 In the following sections, “the concept” will be used as a shorthand for the concept of the interdisciplinary medical group practice.

3 Over time, their work was increasingly shaped by political and socioeconomic developments outside of their immediate control: contrary to their West German colleagues, not as an overabundance of technology but rather a lack of tools and equipment.

4 The model of the group practice had already existed briefly in the Weimar Republic of the early 20^th^ century in the form of joint *Gesundheitszentren* (‘health centers’) or *Ambulatorien*, in which physicians from different medical disciplines and specialties worked together under one roof. However, these were mostly abolished under Nazi rule (Wolff 1997).

5 “Critical” initiatives also existed for other health-related fields and professions, e.g. the *Arbeitskreis Kritische Schwestern* (‘Working Group Critical Nurses’), although there is hardly any literature available on their activities (Herrmann 1969).

6 These publications formed an essential part of the increasingly porous border between academic and public discourse around medicine, producing the sort of critical “counter-knowledge” (*Gegenwissen*) (von Schwerin 2022) or “knowledge from below” (Stadler et al. 2020) constitutive of many social movements at the time. Some examples were *Kritische Krankenhauspresse* (‘Critical Hospital Press’) in Berlin, *Sectio* in Frankfurt, *Zeitung für gesellschaftsbezogene Medizin und herrschaftsfreie Zusammenarbeit im Gesundheitswesen* (‘Newspaper for Society-Oriented Medicine and Non-Hierarchical Cooperation in Healthcare’) in Mainz, or the evocative *Rote Spritze* (‘Red Syringe’) in Homburg/Saar.
